# Relationship between serum 25-hydroxyvitamin D and target organ damage in children with essential hypertension

**DOI:** 10.1038/s41371-021-00622-4

**Published:** 2022-01-09

**Authors:** Yang Liu, Lin Shi, Yao Lin, Mingming Zhang, Fangfang Chen, Aijie Li, Yanyan Liu

**Affiliations:** 1 Department of Cardiology, Children’s Hospital Affiliated to Capital Institute of Pediatrics, 100020 Beijing, China; 2 Department of Epidemiology, Capital Institute of Pediatrics, 100020 Beijing, China

**Keywords:** Clinical trials, Hypertension

## Abstract

Researchers have shown that 25-hydroxyvitamin D (25[OH] D), a kind of active vitamin D in the human body, plays a role in cardiovascular disease (CVD). Low serum 25(OH) D levels have been found to be associated with elevated blood pressure (BP) in adults. However, measurement of 25(OH) D in hypertensive children has not been documented. The aim of this study was to investigate the relationship between 25(OH) D and target organ damage (TOD) in children with essential hypertension. We recruited a total of 346 children with essential hypertension and analyzed the correlation between serum 25(OH) D and TOD. Serum 25(OH) D concentration was significantly lower in the TOD than in the no-TOD group (*t* = 2.416, *P* = 0.016), as well as significantly lower in the two-organ damage than in the single-organ damage group (*t* = 3.140, *P* = 0.002). Pearson’s correlation coefficient (PCC) indicated that serum 25(OH) D levels were negatively correlated with left ventricular mass index (LVMI; *r* = −0.110, *P* = 0.041) and albuminuria (*r* = −0.120, *P* = 0.026). Linear- regression analysis showed that 25(OH) D was a risk factor for left ventricular hypertrophy (LVH; *β* ± *s.e. =*−0.074 ± 0.036; 95% confidence interval [CI], − 0.145 to –0.003; *P* < 0.001) and renal damage (*β* ± *s.e.= −*0.018 ± 0.008; 95% CI, − 0.035 to –0.002; *P* = 0.004). In total, our data revealed that serum 25(OH) D was independently associated with hypertensive cardiac and renal damage, meaning that it was a risk factor for LVH and albuminuria in childhood hypertension.

## Introduction

In recent years, the incidence of essential hypertension in children and adolescents has increased. From 1991 to 2009, the prevalence of hypertension in children and adolescents ages 6–17 years increased by an average of 0.47% annually in China. By 2009, the prevalence of hypertension in children and adolescents in China reached 13.8% [1].

Hypertension is a disease that seriously threatens human health by causing damage to multiple target organs. Hypertension in children and adolescents can cause target organ damage (TOD) to the cardiovascular (CV) system, kidneys, central nervous system (CNS) and fundus. Studies have found that vitamin D receptors are widely present in organs and tissues such as the heart, kidneys and vascular endothelium, which suggests that in addition to its well-known biological effects on bone, vitamin D might also participate in the regulation of CV metabolism [2]. Previous studies have found that low serum vitamin D is a risk factor for CV disease (CVD) and is associated with increased incidence of hypertension, insulin resistance, obesity, metabolic syndrome, impaired fasting blood glucose (FBG) levels and dyslipidemia [3, 4]. In China, a prospective cohort study on the associations between vitamin D nutritional status and cardiometabolic abnormities in children showed that the vitamin D deficiency rate was 35.1% and the 2-year cumulative incidence rate of hypertension was 10.8% [5]. Vitamin D exerts its regulatory effect on BP through various mechanisms: it confers a renoprotective effect and lowers BP by inhibiting renin secretion and the renin– angiotensin system (RAAS) [6], improves vascular endothelial function [7], and reduces vascular oxidative stress (OS) [8]. In our previous studies [9], we found that abnormal activation of RAAS was associated with TOD in children with essential hypertension, plasma angiotensin II (AT II) and aldosterone levels were associated with cardiac damage, and AT II and aldosterone were risk factors for left ventricular hypertrophy (LVH) in childhood hypertension. As a kind of active vitamin D in the human body, 25(OH) D is recognized as the best index to evaluate vitamin D status. Earlier studies have shown that vitamin D deficiency is independently related to TOD in adults with essential hypertension [10]. However, the relationship of 25(OH) D levels to BP levels and TOD in hypertensive children remains unclear. Therefore, we conducted a correlation analysis between serum 25(OH) D and BP levels as well as TOD in children with essential hypertension from June 2016 to March 2021.

## Methods

### Subjects

This study was conducted single-center and cross-sectional. We recruited a total of 346 children, including 275 males (79.5%) and 71 females (20.5%), diagnosed with essential hypertension and admitted to the Affiliated Children’s Hospital of Capital Institute of Pediatrics (CIP; Beijing, China) from June 2016 to March 2021. The research protocol of this study was approved by the Ethics Committee of the CIP. Patients’ parents or guardians of the patients received information about the relevant examinations and signed the written informed consent beforehand.

We performed all BP measurements using the auscultation method as recommended in *The Fourth Report on the Diagnosis, Evaluation, and Treatment of High Blood Pressure in Children and Adolescents* and used the results to diagnose hypertension and perform stage classification [11]. Hypertension was diagnosed when the average systolic and/or diastolic BP was in the ≥95th percentile of the auscultation measurement on at least three separate occasions, adjusted for gender, age, and height. Stage 1 hypertension was diagnosed if a child’s BP was greater than the 95th percentile but less than or equal to the 99th percentile plus 5 mmHg, and stage 2 hypertension was diagnosed if a child’s BP was greater than the 99th percentile plus 5 mmHg.

Exclusion criteria for this study were patients >18 years; with secondary hypertension caused by kidney disease, vascular disease, endocrine disease, CNS disease or drugs; or systemic diseases with abnormal calcium and phosphorus metabolism.

### Laboratory examinations

#### Blood lipids, electrolytes and other biochemical-indicator measurements

Pediatric patients fasted overnight for 8 h, In the morning, we collected their venous blood to measure serum sodium, potassium and calcium concentrations; plasma cholesterol; triglyceride; uric acid and other parameters. All biochemical variables were assayed in plasma or serum using standard methods.

#### Serum 25(OH) D concentration measurements

We determined vitamin D content in serum by detecting 25(OH) D in serum. Venous-blood samples were taken from patients in the morning after 12 h of overnight fasting. After centrifugation for 15 min (2000 g), we collected the samples in tubes and refrigerated them. Concentrations of 25(OH) D were assessed using a chemiluminescence immunoassay (Immunodiagnostic Systems [IDS] Holdings Ltd, Tyne & Wear, UK). The limit of detection (LoD) for 25(OH) D was 4.8 ng/mL; no samples had concentrations below LoD. All samples were run in duplicate and the values averaged. Total (intra-assay and inter-assay) coefficients of variation for control values of 12.0 and 77.9 ng/mL were 10.0% and 7.6%, respectively.

We classified the status of 25(OH) D concentration as deficient (<30 nmol/L), inadequate (30 to <50 nmol/L), or adequate (≥50 nmol/L) per the report released in 2010 by the Institute of Medicine [12]. The timing of blood sampling was classified by Northern Hemisphere seasons: spring (March to May), summer (June to August), autumn (September to November) or winter (December to February).

#### Ambulatory blood pressure measurement

All patients underwent 24-h ambulatory BP monitoring via a DM Software Ambulatory Blood Pressure (DMS-ABP) device (DM Software, Inc., Beijing, China). They received information on the procedure and device beforehand. In order for the device to function properly, patients were told to perform their daily activities normally but to remain immobile during measurements. BP recordings were programmed to occur every 30 min during the day and every 60 min while patients slept. We recorded and adjusted sleep and wake times for each patient to define the nighttime period. Based on these measurements, we determined 24- h systolic BP (24-h SBP), 24-h diastolic BP (24-h DBP), daytime SBP, daytime DBP, nighttime SBP and nighttime DBP. The method was considered reliable if >75% of the measurements were valid.

### Indicators for evaluating target organ damage

#### Heart

We assessed LVH via echocardiography (ECG). Left ventricular internal dimension (LVIDd), interventricular septal thickness (IVST) and left ventricular posterior-wall thickness (LVPWT) at the end diastole were measured using a Philips iE33 Ultrasound System (Philips Healthcare, Bothell, WA, USA). Left ventricular mass (LVM) was calculated as follows:

$${{{{{{{\mathrm{LVM}}}}}}}} \!=\! 1.04 \times 0.8 \times ( {[ {{{{{{{{\mathrm{LVIDd}}}}}}}} + {{{{{{{\mathrm{IVST}}}}}}}} + {{{{{{{\mathrm{LVPWT}}}}}}}}} ]^3-{{{{{{{\mathrm{LVIDd}}}}}}}}^3} ) \!+\! 0.6$$ [13].

LVM index (LVMI) was calculated as LVM/height^2.16^. Relative left ventricular wall thickness (RWT) was calculated as (IVST + LVPWT)/LVIDd. For the diagnosis of LVH, LVMI ≥ 45 g/m^2.16^ or RWT > 0.41 was considered abnormal [14, 15].

#### Kidneys

We diagnosed patients with renal damage if any of the following conditions was fulfilled: albuminuria (urinary albumin/creatinine [Cr] quotient) >3 mg/mmol Cr; 24-h urine protein ^excretion^>200 mg/m^2^/day; urine β_2_-microglobulin (β_2_- MG) > 0.3 mg/L or cystatin C (Cys-C) > 1.26 mg/L [16–19].

#### Groupings

We grouped patients with essential hypertension by 25(OH) D level: a deficiency group (<30 nmol/L), an inadequacy group (30 to <50 nmol/L), and an adequacy group (≥50 nmol/L). We also grouped patients by presence or absence of TOD; patients in the TOD group were further subdivided into cardiac-damage and renal-damage groups. We then analyzed differences in serum 25(OH) D levels among these groups.

### Statistical methods

We used SPSS software version 20.0 (IBM Corp., Armonk, NY, USA) to process all data in this study. Values of parameters with normal distribution were presented as means ± standard deviations (SDs), whereas values with non-normal distributions were transformed into normal distributions by logarithmic transformation. We used an independent-sample *t* test to compare differences between two groups. Analysis of variance (ANOVA) was used to compare measured differences among multiple groups. Count data are presented as percentages (%). Univariate associations between the indices of 25(OH) D levels, LVMI, and other variables of TOD were assessed using simple linear-regression analyses and Pearson’s correlation coefficient (PCC). *P* < 0.05 was considered statistically significant.

## Results

### Demographics, laboratory characteristics and ambulatory blood pressure measurements

Among the 346 children with essential hypertension enrolled in this study, there were 275 males (79.5%) and 71 females (20.5%), with a mean age of 12.3 ± 2. 2 years (12.2 ± 2. 2 years for males, 12.7 ± 2.2 years for females), average height of 16 6.3 ± 13.2 cm, average body weight of 75.2 ± 20.2 kg and average body mass index (BMI) of 26.8 ± 5. 1 kg/m^2^. Of these, 253 patients (73.1%) had a BMI exceeding the 95th percentile of children of the same age and gender and were diagnosed as obese. Average 24-h SBP and 24-h DBP of the 346 patients were 126.9 ± 10.3 mmHg and 71.3 ± 6. 9 mmHg, respectively. All 346 were treated at our hospital and had received no previous drug interventions.

Demographic and laboratory characteristics and ambulatory BP measurements are shown in Table 1. Of the total 346 patients, 62 (17.9%) presented vitamin D adequacy, 166 (48.0%) presented inadequacy, and 118 (34.1%) presented deficiency. Comparisons of clinical and laboratory characteristics and BP measurements, including gender, BMI, blood lipids, serum uric acid, glucose, serum sodium, potassium, calcium and phosphorus concentrations, 24-h SBP and DBP; daytime SBP and DBP; nighttime SBP and DBP; and systolic and diastolic dipping ratios, showed no significant differences among the vitamin D adequacy, vitamin D inadequacy and vitamin D deficiency groups. Age comparisons across all three groups showed that differences in age were statistically significant (*P* < 0.001).

**Table 1:** Comparison of demographic, laboratory characteristics, and ambulatory blood pressure measurements in different groups of serum 25 (OH) D levels.

Parameters	Vitamin D adequacy group (*n* = 62)	Vitamin D inadequacy group (*n* = 166)	Vitamin D deficiency group (*n* = 118)	*P* value
Age (year)	11.66 ± 2.35	12.05 ± 2.32	12.97 ± 1.88	<0.001*
Sex (male)	57 (91.9%)	131 (78.9%)	89 (75.4%)	0.108
BMI (Kg/m^2^)	26.07 ± 4.42	26.71 ± 4.90	27.18 ± 5.66	0.372
CHOL (mmol/L)	4.01 ± 0.75	3.91 ± 0.68	3.91 ± 0.66	0.599
Season of blood collection, *n* (%)				
Spring	4 (6.5)	28 (16.9)	43 (36.4)	
Summer	36 (58.1)	45 (27.1)	9 (7.6)	
Autumn	14 (22.6)	51 (30.7)	13 (11.0)	
Winter	8 (12.9)	42 (25.3)	53 (44.9)	
TG (mmol/L)	1.26 ± 0.54	1.27 ± 0.63	1.25 ± 0.59	0.948
UA (μmol/L)	430.02 ± 85.86	412.58 ± 103.36	438.58 ± 95.97	0.080
Glucose(mmol/L)	4.51 ± 0.45	4.59 ± 0.60	4.50 ± 0.49	0.362
Blood sodium (mmol/L)	141.40 ± 1.54	141.86 ± 1.63	141.69 ± 1.84	0.196
Blood potassium (mmol/L)	4.26 ± 0.41	4.24 ± 0.38	4.15 ± 0.30	0.054
Blood calcium (mmol/L)	2.43 ± 0.16	2.42 ± 0.16	2.43 ± 0.16	0.889
Blood phosphorus (mmol/L)	1.82 ± 0.20	1.80 ± 0.16	1.77 ± 0.19	0.178
24-h SBP (mmHg)	126.2 ± 10.5	126.5 ± 10.2	128.0 ± 10.3	0.396
24-h DBP (mmHg)	70.3 ± 7.3	71.1 ± 6.8	72.0 ± 6.9	0.258
Daytime SBP (mmHg)	128.0 ± 11.6	128.9 ± 10.8	130.1 ± 11.1	0.420
Daytime DBP (mmHg)	72.8 ± 9.9	73.3 ± 7.4	73.7 ± 7.5	0.743
Nighttime SBP (mmHg)	121.6 ± 13.0	122.7 ± 10.4	123.1 ± 10.2	0.646
Nighttime DBP (mmHg)	67.6 ± 7.7	68.1 ± 7.1	68.0 ± 8.0	0.893
Systolic dipping ratio (%)	4.1 ± 5.2	4.6 ± 5.0	5.0 ± 4.8	0.518
Diastolic dipping ratio (%)	6.7 ± 8.1	6.8 ± 7.5	7.5 ± 9.1	0.722

*BMI* body mass index, *CHOL* cholesterol, *TG* triglyceride, *UA* uric acid, *SBP* systolic blood pressure, *DBP* diastolic blood pressure. *P < 0.05 is considered for statistical analysis.

### Evaluation of target organ damages

We evaluated all 346 children for heart and kidney damage. Of all 346 cases, 179 (51.7%) had TOD, including 138 patients with a single organ damaged and 41 patients with two organs damaged.

#### Heart

Ninety-four (27.2%) of the 346 patients had LVH. Of these, 81 had increased LVMI and normal RWT, manifesting as eccentric remodeling; 8 had normal LVMI and increased RWT, with concentric remodeling; and 5 had increased LVMI and RWT, manifesting as concentric hypertrophy.

#### Kidneys

One hundred twenty-six (36.4%) of the 346 patients had renal damage, 10 (2.9%) had elevated 24-h urine protein, 60 (17.3%) had albuminuria, 58 (16.8%) had elevated urine β2- MG and 27 (7.8%) had increased Cys-C.

#### Relationship between serum 25(OH) D levels and blood pressure levels

Of all 346 patients, 183 (52.9%) had stage 1 hypertension, and 163 (47.1%) had stage 2 hypertension. There was no significant difference in serum 25(OH) D concentrations between these two groups (38.48 ± 12.87 nmol/L *vs*. 36.99 ± 13. 59 nmol/L; *t* = 1.048, *P* = 0.295).

#### Relationship between serum 25(OH) D levels and target organ damage

We compared baseline characteristics between the TOD and no-TOD groups. Of all 346 patients, 179 (51.7%) had TOD, including 148 males (82.7%) and 31 females (17.3%), with a mean age of 12.4 ± 2. 1 years; 167 patients (48.3%) had no TOD, including 127 males (76.0%) and 40 females (24.0%), with a mean age of 12.2 ± 2.4 years. No signifi cant differences in sex or age were found between these two groups (*χ*^*2*^ = 2.333, *P* = 0.128; *t* = 0.925, *P* = 0.356).

Serum 25(OH) D concentration in the TOD group (lg10, 1.53 ± 0.14 nmol/L) was significantly lower than in the no-TOD group (lg10, 1.57 ± 0.15 nmol/L), and the difference was statistically significant (*t* = 2.416, *P* = 0.016). In the TOD group, 138 cases had single- organ damage, while 41 cases had simultaneous two-organs damage. Serum 25(OH) D concentrations of the two groups were lg10, 1.55 ± 0.15 nmol/L and lg10, 1.47 ± 0.10 nmol/L, respectively; serum 25(OH) D levels in the two-organs damage group were significantly lower than in the single organ damage group (*t* = 3.140, *P* = 0.002).

**Fig. 1:**
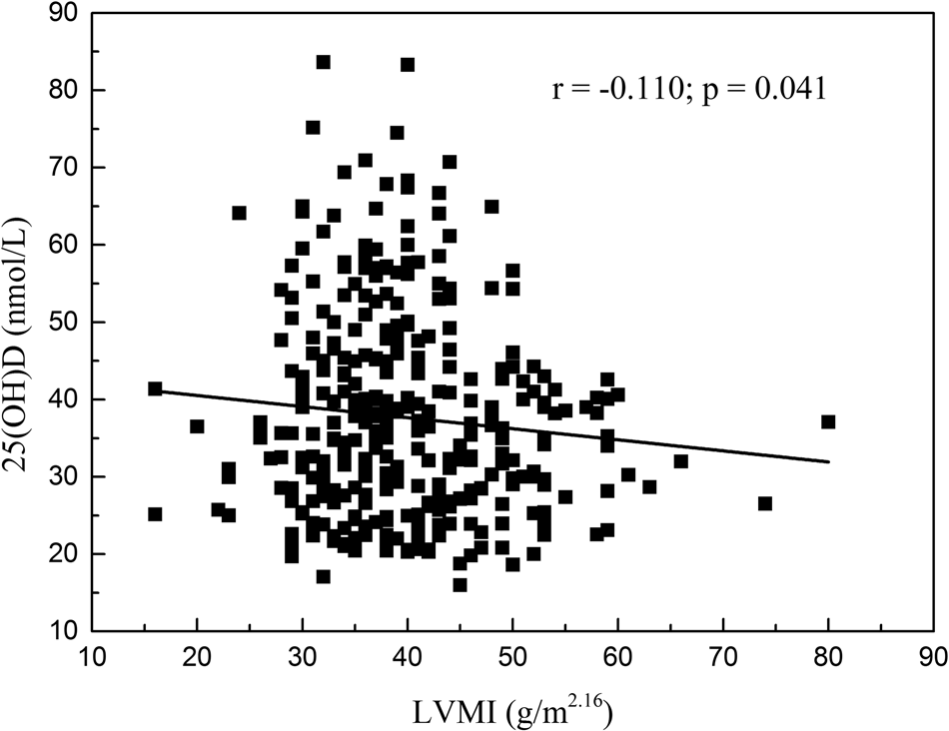
Correlations between serum 25(OH) D levels and LVMI. Pearson’s correlation coefficient indicated that correlations between serum 25(OH) D levels and LVMI were negative (*r* = −0.110, *P* = 0.041).

**Fig. 2:**
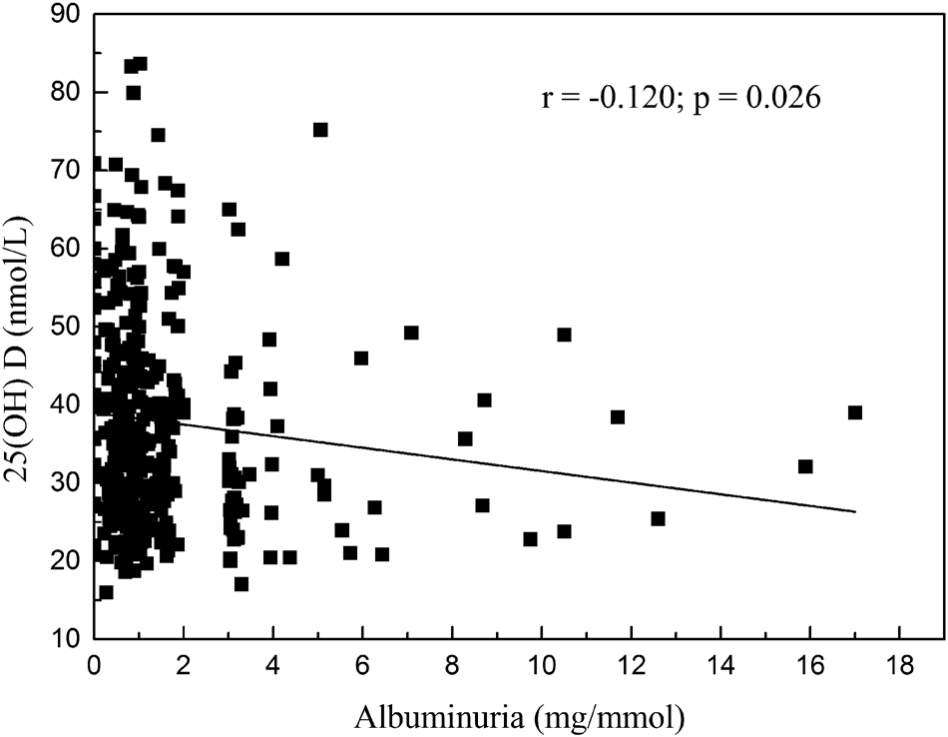
Correlations between serum 25(OH) D levels and albuminuria. Pearson’s correlation coefficient indicated that correlations between serum 25(OH) D levels and albuminuria were negative (*r* = −0.120, *P* = 0.026).

We analyzed the correlation between serum 25(OH) D levels and TOD in all 346 patients. As shown in Table 2, serum 25(OH) D levels were correlated with LVMI and albuminuria (*P* < 0.05); further analysis via PCC indicated that these correlations were negative (LVMI: *r* = −0.110, *P* = 0.041, Fig. 1; albuminuria: *r* = −0.120, *P* = 0.026, Fig. 2). Linear-regression analysis showed that when adjusted by sex, age, BMI, season of blood collection, SBP and DBP, 25(OH) D was a risk factor for LVH (*β* ± s.e. = −0.074 ± 0.036; 95% confidence interval [CI], − 0.145 to –0.003; *P* < 0.001) and renal damage (*β* ± s.e. = −0.018 ± 0.008; 95% CI, − 0.035 to –0.002; *P* = 0.004; Table 3). There were no significant correlations between 25(OH) D levels and other indicators, such as RWT, urine β2-MG or Cys-C (*β* ± s.e. < 0.001).

**Table 2:** Correlation analysis between serum 25(OH) D levels and target organ damages.

Target organ damage	*r-*value	*P* value
LVMI	−0.110	0.041*
RWT	−0.010	0.869
Albuminuria	−0.120	0.026*
β_2_⁃MG	−0.071	0.185
Cys-C	−0.020	0.715

*LVMI* ventricular mass index, *RWT* relative wall thickness, *β*_*2*_⁃*MG* β_2_⁃microglobulin, *Cys-C* Cystatin C.

*P < 0.05 is considered for statistical analysis.

**Table 3:** Linear regression analysis between serum 25(OH) D levels and target organ damages.

Characteristic	*β* ± *s.e*.	*95% CI*		*P* value
LVMI	−0.074 ± 0.036 R^2^ = 0.260	−0.145	−0.003	<0.001*
Albuminuria	−0.018 ± 0.008 R^2^ = 0.040	−0.035	−0.002	0.004*

Adjusted by sex, age, BMI, season of blood collection, SBP, and DBP.

*LVMI* ventricular mass index, *95% CI* 95% confidence interval.

*P < 0.05 is considered for statistical analysis.

## Discussion

Essential hypertension is a multi-gene disease in which the genetic components interact with physiological factors. Recent research has shown vitamin D receptors to be widely distributed throughout almost all the organs of the body [20], and vitamin D to be related to chronic diseases such as CVD, diabetes, and cancer [21]. In addition, 1, 25-dihydroxyvitamin D3 (1,25[OH]2D3) has been found to directly or indirectly regulate a variety of genes [22], such as those related to CVD, and to participate in the development of CVD [23]. However, relatively few studies exist on the relationship between serum 25(OH) D concentration and BP levels, as well as on TOD in children with essential hypertension.

Several studies have proven a strong relationship between vitamin D deficiency and TOD [24]. Vitamin D exerts CV-protective effects by modulating inflammatory cytokines, OS and systemic RAAS [25]. First, experimental studies have shown that 1,25(OH)2D3 exerts in vitro anti-hypertrophic effects on cardiomyocytes and reduces the expression of several genes that are upregulated in myocardial hypertrophy; suppression of the cardiac RAAS and natriuretic peptides might partially mediate these anti-hypertrophic effects [26, 27]. Second, vitamin D deficiency triggers secondary hyperparathyroidism; parathyroid hormone (PTH) promotes myocytic hypertrophy and vascular remodeling. Other studies suggest that PTH has a pro-inflammatory effect, which can stimulate the release of cytokines by vascular smooth- muscle cells (VSMCs) [28, 29]. Third, lower serum 25(OH) D levels result in RAAS hyperactivity, which can damage vasculature and stiffen arteries [30]. Moreover, vitamin D suppresses proliferation of VSMCs and activation of macrophages [31, 32]. Finally, early vitamin D deficiency can increase arterial BP, damage tissue cells and eventually lead to a change in cardiac-gene expression [8]. Our results showed that serum 25(OH) D concentration in the TOD group was significantly lower than in the no-TOD group.

Therefore, serum 25(OH) D concentration correlated negatively with TOD, the incidence of which was higher in children with low serum 25(OH) D levels.

Myocardial remodeling in hypertension refers to cardiac hypertrophy and fibrosis. Sustained BP load first and mainly affects the left heart, resulting in LVH. LVMI and RWT are important indices by which to evaluate early LVH. LVH is often the main manifestation of early cardiac damage in children with hypertension. As an intermediate stage of heart damage during the progression from childhood to adulthood, early heart damage is important in evaluations. Not only is LVH related to BP values [33], but current studies also point out that vitamin D deficiency might be the influencing factor in cardiac hypertrophy [34]. Our study analyzed the correlation between 25(OH) D levels and LVMI and showed an association, which we further evaluated using linear-regression analysis. The results showed that serum 25(OH) D levels in patients with LVH were lower than in patients without LVH. After adjusting for gender, age, BMI, season of blood collection, SBP and DBP, we found that serum 25(OH) D was negatively correlated with LVMI and was a risk factor for LVH in hypertensive patients.

The kidney is one of the main target organs in patients with hypertension. Owing to the hidden onset of hypertension and the strong compensatory ability of the kidneys, some hypertensive children with kidney damage show no obvious abnormalities in routine renal-function monitoring indicators. Albuminuria, urine β2-MG and Cys-C are sensitive indicators reflecting early hypertensive renal damage, suggesting impaired glomerular-filtration function and proximal convoluted tubular-reabsorption function [35]. In our study, we also found that renal involvement in children with essential hypertension was mainly caused by significant increases in albuminuria, urine β2-MG and Cys-C. When we analyzed the correlation between serum 25(OH) D levels and renal damage in 346 children with hypertension, we found it to be negative. We used PCC to evaluate the correlation between each index of early renal damage and 25(OH) D level, and the results showed a negative correlation between serum 25(OH) D concentration and albuminuria. Serum 25(OH) D levels of patients with essential hypertension complicated by early renal damage were lower than in patients without renal damage. Therefore, serum 25(OH) D was related to the incidence of early renal damage, consistent with the study on adult hypertension [10].

In conclusion, 25(OH) D deficiency was a frequent finding in children who had essential hypertension without major CV risk factors. Our results showed that serum 25(OH) D levels were independently associated with hypertensive cardiac and renal damages and that low serum 25(OH) D was a risk factor for LVH and albuminuria in childhood hypertension.

Some potential limitations of the present study should be discussed. First, considering that this was a small-scale study from a single center, the generalizability of our data might be limited. The low number of females might also limit its statistical power. Second, carotid intima-media thickness (CIMT) and pulse wave velocity (PWV) are also considered markers of TOD, but we did not assess them in this study, which is another limitation of the study.

Third, several studies have shown that serum/plasma levels of PTH are positively associated with increased BP [36]. However, in our study, we did not investigate the correlation of PTH with BP level and TOD in children with essential hypertension. Finally, because our study design was cross- sectional, we did not provide any intervention to children with vitamin D inadequacy or deficiency. Vitamin D therapy is necessary for children who manifest clinical features of hypocalcemia as a result of vitamin D deficiency and when vitamin D levels are in the deficient range [37]. These children should be further evaluated over the long term, and their 25(OH) D levels in particular should be monitored, to more fully discern the effect of serum 25(OH) D on BP levels and TOD.

## Data Availability

The raw data supporting the conclusions of this article will be made available by the authors, without undue reservation.
